# Impact of Educational Attainment on Health Outcomes in Moderate to Severe CKD

**DOI:** 10.1053/j.ajkd.2015.07.021

**Published:** 2016-01

**Authors:** Rachael L. Morton, Iryna Schlackow, Natalie Staplin, Alastair Gray, Alan Cass, Richard Haynes, Jonathan Emberson, William Herrington, Martin J. Landray, Colin Baigent, Borislava Mihaylova, SHARP Collaborative Group

**Affiliations:** 1 NHMRC Clinical Trials Centre, The University of Sydney, Sydney, Australia; 2 Health Economics Research Centre, University of Oxford, Oxford, United Kingdom; 3 Clinical Trial Service Unit and Epidemiological Studies Unit, Nuffield Department of Population Health, University of Oxford, Oxford, United Kingdom; 4 Menzies School of Health Research, Charles Darwin University, Darwin, Australia

**Keywords:** Chronic kidney failure, chronic kidney disease (CKD), education, educational attainment, disease progression, end-stage renal disease (ESRD), vascular event, mortality, health behavior, risk factor, socioeconomic factors, inequalities, renal dialysis, Study of Heart and Renal Protection (SHARP)

## Abstract

**Background:**

The inverse association between educational attainment and mortality is well established, but its relevance to vascular events and renal progression in a population with chronic kidney disease (CKD) is less clear. This study aims to determine the association between highest educational attainment and risk of vascular events, cause-specific mortality, and CKD progression.

**Study Design:**

Prospective epidemiologic analysis among participants in the Study of Heart and Renal Protection (SHARP), a randomized controlled trial.

**Setting & Participants:**

9,270 adults with moderate to severe CKD (6,245 not receiving dialysis at baseline) and no history of myocardial infarction or coronary revascularization recruited in Europe, North America, Asia, Australia, and New Zealand.

**Predictor:**

Highest educational attainment measured at study entry using 6 levels that ranged from “no formal education” to “tertiary education.”

**Outcomes:**

Any vascular event (any fatal or nonfatal cardiac, cerebrovascular, or peripheral vascular event), cause-specific mortality, and CKD progression during 4.9 years’ median follow-up.

**Results:**

There was a significant trend (*P* < 0.001) toward increased vascular risk with decreasing levels of education. Participants with no formal education were at a 46% higher risk of vascular events (relative risk [RR], 1.46; 95% CI, 1.14-1.86) compared with participants with tertiary education. The trend for mortality across education levels was also significant (*P* < 0.001): all-cause mortality was twice as high among those with no formal education compared with tertiary-educated individuals (RR, 2.05; 95% CI, 1.62-2.58), and significant increases were seen for both vascular (RR, 1.84; 95% CI, 1.21-2.81) and nonvascular (RR, 2.15; 95% CI, 1.60-2.89) deaths. Lifestyle factors and prior disease explain most of the excess mortality risk. Among 6,245 participants not receiving dialysis at baseline, education level was not significantly associated with progression to end-stage renal disease or doubling of creatinine level (*P* for trend = 0.4).

**Limitations:**

No data for employment or health insurance coverage.

**Conclusions:**

Lower educational attainment is associated with increased risk of adverse health outcomes in individuals with CKD.

**Editorial,** **p. 1**

The inverse association between education and health outcomes is well established in the general population, with a gradient in health across levels of educational attainment.^1^ Two general mechanisms, lifestyle factors (or behaviors) and access to effective health care, are believed to contribute to this gradient.^1^, ^2^, ^3^, ^4^, ^5^ Previous work has shown that health behaviors explain some of the observed changes in the education-mortality gradient over time across the population,^6^ with lower levels of smoking and better control of hypertension associated with lower age-adjusted mortality, and increased obesity associated with higher mortality risk.^6^

However, studies of the relevance of educational attainment to health outcomes in chronic kidney disease (CKD) are sparse.^7^ Two small studies of patients on dialysis therapy have shown that poor health literacy (defined as the ability to obtain, process, and understand basic health information to make decisions regarding one’s health and medical care) is associated with 54% (hazard ratio [HR], 1.54; 95% confidence interval [CI], 1.01-2.35) higher risk of death,^8^ and tertiary education compared with primary school education only is associated with 46% (HR, 0.54; 95% CI, 0.32-0.91) lower cardiovascular mortality.^9^

We used data from 9,270 participants with moderate to severe CKD in the Study of Heart and Renal Protection (SHARP) to: (1) assess associations between highest educational attainment (ie, level of schooling, vocational, or tertiary education obtained early in life) and particular health outcomes (vascular morbidity, cause-specific mortality, and kidney disease progression) and (2) determine whether there are gradients in health outcomes by educational attainment in moderate to severe CKD. With more than 2,300 vascular events, more than 2,200 deaths, and more than 2,400 participants doubling their creatinine levels or progressing to end-stage renal disease (ESRD) during a median 4.9 years’ follow-up, the study provides an opportunity to investigate associations between educational attainment and health outcomes in a large and carefully phenotyped population with CKD.

## Methods

### Study Overview

SHARP was a randomized placebo-controlled trial investigating the effects of lowering low-density lipoprotein cholesterol levels for an average of 5 years with simvastatin, 20 mg, plus ezetimibe, 10 mg, daily on major vascular and kidney disease outcomes. The study enrolled 9,270 participants with CKD (6,245 [67%] not receiving and 3,025 [33%] receiving dialysis at baseline) in 18 countries.^10^ Study procedures and clinical results from the randomized comparisons have been published previously.^10^, ^11^ Procedures for the current analyses followed STROBE (Strengthening the Reporting of Observational Studies in Epidemiology) guidelines^12^ and are summarized next.

### Study Participants and Baseline Assessment

Individuals with CKD who were 40 years or older were eligible to participate if they had more than one previous measurement of serum or plasma creatinine of at least 1.7 mg/dL (150 μmol/L) in men or 1.5 mg/dL (130 μmol/L) in women. Individuals with prior myocardial infarction or coronary revascularization and those with a medical history that might limit their ability to participate or take study treatments for the duration of the study (eg, severe respiratory disease, history of cancer other than nonmelanoma skin cancer, or recent history of alcohol or substance misuse) were excluded. A 6-week run-in period was undertaken to identify and exclude from randomization participants who were unlikely to adhere to the study medication for the duration of the study. For the purpose of the current analyses, baseline information refers to information that was recorded at or shortly before a participant’s random assignment to simvastatin plus ezetimibe versus placebo. Recorded baseline information included sociodemographic characteristics (such as age, sex, and ethnicity), blood pressure, blood and urine test results, anthropometric measurements to calculate body mass index (BMI), previous disease history, current medications, cigarette smoking, and alcohol consumption. To facilitate the planned analysis of the impact of educational attainment on health outcomes, the participant’s highest educational level completed was recorded at trial entry according to the relevant description in each country: postgraduate tertiary education, undergraduate tertiary education, high school, vocational studies, lower high school, primary school, or no formal education (see separate country-specific classifications in Table S1, available as online supplementary material). In addition, participants without recorded education data were categorized as “unrecorded.” Due to the small number of participants with postgraduate tertiary education, the top 2 education categories were combined to make one tertiary education level. Estimated glomerular filtration rate (eGFR) was calculated using the CKD-EPI (CKD Epidemiology Collaboration) creatinine equation.^13^

### Follow-up Procedures and Study Outcomes

Participants were to be seen in person at 2, 6, and 12 months and every 6 months thereafter for at least 4 years. At each visit, information for all serious adverse events (including vascular events, revascularization procedures, and initiation of renal replacement therapy), adherence to study medication, and use of concomitant medication was collected; further information was also sought from hospital and other health records. Trained clinicians at the international coordinating center adjudicated major study outcomes, including cause-specific mortality, using standardized definitions and procedures.

For current analyses, the main vascular outcome of interest is “any vascular event” during the study, defined as nonfatal myocardial infarction, coronary death, hemorrhagic and nonhemorrhagic stroke, arterial revascularization, noncoronary cardiac death; atherosclerotic other coronary, other cerebrovascular, other peripheral arterial disease events; and nonfatal nonischemic heart failure, arrhythmias, or valvular heart disease (see Item S1). Mortality was subdivided into vascular and nonvascular (ie, renal, cancer, respiratory, or other nonvascular) causes. To further understand the possible relevance of educational attainment to respiratory and cancer deaths, exploratory analyses of cancer incidence and nonfatal respiratory events were undertaken. Among participants not receiving dialysis at baseline, the main kidney disease outcomes in this analysis included 2 composite end points: (1) progression to ESRD (defined as kidney transplantation or initiation of maintenance dialysis therapy) or doubling of creatinine level and (2) progression to ESRD or death. In addition, the availability of serial creatinine measurements allowed for estimation of each individual’s annual rate of change in eGFR over time.^14^

### Statistical Analysis

The relevance of highest educational attainment to first occurrence of disease outcomes of interest was estimated using Cox proportional hazards models stratified by country of recruitment. The proportional hazard assumption was tested through examination of the time-dependency of the Schoenfeld residuals.^15^ The gradient in health outcomes across all education levels, before and after adjustment for potential mediators (see text that follows), was estimated using Wald χ^2^ tests for trend after excluding participants for whom education was unrecorded. The χ^2^ statistics allow for a quantitative assessment of both the extent to which the mediators explain any education gradients and the residual (ie, unexplained) relevance of education to risk after adjustment for the recorded mediators.

Linear regression models were used to assess the relevance of education level to rate of decline in eGFR. All analyses were adjusted for age, sex, ethnicity (black vs white/other), and allocation to simvastatin plus ezetimibe (vs placebo) and stratified by country of recruitment. The relationship between educational attainment and health outcomes was compared in the 2 treatment groups (simvastatin plus ezetimibe vs placebo) with a test for heterogeneity. Models for estimating total effects of education on health outcomes did not adjust for the following variables because they were assumed to be on the causal pathway between educational attainment and the outcomes of interest: cigarette smoking, current alcohol consumption, BMI, comorbid conditions (vascular disease and diabetes mellitus), CKD stage, renal diagnosis, systolic blood pressure, diastolic blood pressure, albumin level, urinary albumin-creatinine ratio, hemoglobin level, phosphate level, high-density lipoprotein cholesterol level, and total cholesterol level (Fig S1).^16^ Definitions for model covariates are provided in Item S2. Cox proportional hazards models were compared before and after inclusion of all available potential effect mediators to examine how the relationships observed across education levels depended on these variables and to assess the extent to which the association was explained by them. Associations between educational level and behavioral risk factors (smoking, obesity [BMI > 30 kg/m^2^], alcohol use, and adherence to allocated study treatment [simvastatin plus ezetimibe or placebo]) were assessed using logistic regression models stratified by country or region of recruitment.^17^

For descriptive statistics, *P* values for differences between education levels (heterogeneity or trend) were calculated using likelihood ratio test statistics from linear regression models for continuous variables and logistic regression models for categorical variables.^18^ In the graphical figures, relative risks (RRs: approximated by the HR estimates from the Cox models) for each education group are presented as “floating absolute risks,” which assign an appropriate variance to the log of the RR in each group, including the reference group of tertiary education.^19^ All RRs quoted in the text are, unless stated otherwise, for each educational level versus tertiary education. STATA, version 13 (StataCorp LP), and SAS, version 9 (SAS Institute Inc), software were used for statistical modeling.

## Results

### Characteristics of Study Participants by Education Level

In SHARP, 9,270 participants from 18 countries were randomly assigned to simvastatin plus ezetimibe versus placebo (mean age 62 years; 63% men; 72% white; 22% Asian; 3% black; and 3% other ethnicity). Of them, 11% completed tertiary education; 14%, high school; 23%, vocational qualifications; 19%, lower high school; and 15%, primary school; 4% completed no formal education, and data were unrecorded for 14% (Table 1). Those with lower educational attainment were more likely to be older, women, from Asia, receiving dialysis, and a current smoker.

### Vascular Events

**Figure 1:**
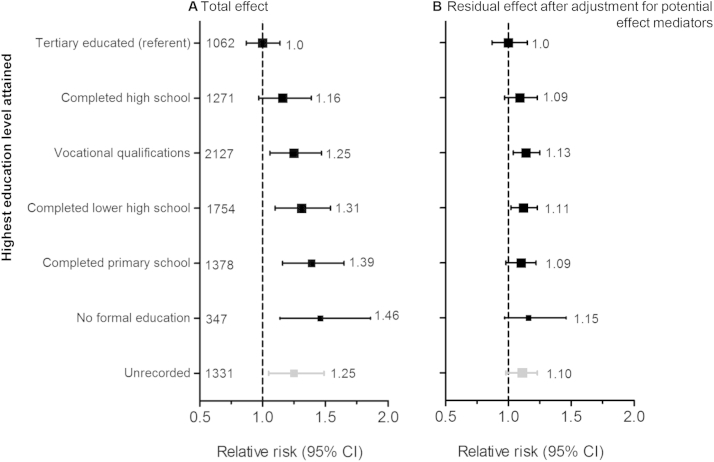
Relevance of highest education level attained to 2,317 vascular events (atherosclerotic and nonatherosclerotic). (A) Total effect: Cox proportional hazards model stratified by country and adjusted for age, sex, black ethnicity, and study treatment assignment. Test for trend χ^2^ = 16.12; *P* < 0.001. (B) Residual effect: Cox proportional hazards model stratified by country and adjusted for age, sex, black ethnicity, smoking, alcohol use, body mass index, chronic kidney disease stage, prior vascular disease, diabetes, renal diagnosis, systolic and diastolic blood pressure, albumin level, urinary albumin-creatinine ratio, hemoglobin level, phosphate level, high-density lipoprotein cholesterol level, and total cholesterol level. The size of the square representing a relative risk is proportional to its inverse variance; error bars represent 95% confidence intervals (CIs). Tests for trend in the models were evaluated after excluding participants with unrecorded education. Test for trend χ^2^ = 0.81; *P* = 0.4.

During a median of 4.9 years’ follow-up, 2,317 participants experienced at least one vascular event (66/1,000 patients per year [ppy]). There was a significant trend toward increasing vascular event risk with lower levels of educational attainment (χ^2^ = 16.12; *P* < 0.001; Fig 1A). Compared with tertiary-educated individuals, vascular event risk was 46% higher among those with no formal education (RR, 1.46; 95% CI, 1.14-1.86). After adjustment for potential effect mediators, including cigarette smoking, alcohol use, obesity, adherence to study medication, CKD stage, and comorbid conditions, the effect of educational attainment on vascular risk was substantially attenuated and the trend was no longer statistically significant (χ^2^ = 0.81; *P* = 0.4; Fig 1B).

### Mortality

**Figure 2:**
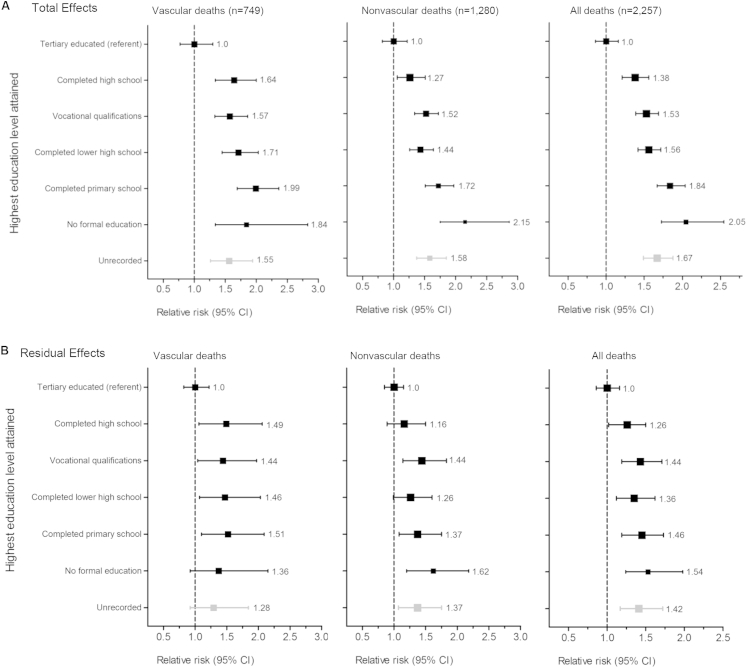
Relevance of highest education level attained to vascular, nonvascular, and overall mortality. (A) Total effects: Cox proportional hazards models stratified by country and adjusted for age, sex, black ethnicity, and study treatment assignment. Tests for trend: χ^2^ = 14.23; *P* < 0.001 for vascular deaths; χ^2^ = 29.42; *P* < 0.001 for nonvascular deaths; χ^2^ = 51.52; *P* < 0.001 for all deaths. (B) Residual effects: Cox proportional hazards models stratified by country and adjusted for age, sex, black ethnicity, study treatment assignment, smoking, alcohol use, body mass index, chronic kidney disease stage, prior vascular disease, diabetes, renal diagnosis, systolic and diastolic blood pressure, albumin level, urinary albumin-creatinine ratio, hemoglobin level, phosphate level, high-density lipoprotein cholesterol level, and total cholesterol level. The size of a square representing a relative risk is proportional to its inverse variance; error bars represent 95% confidence intervals (CIs). Tests for trend in all models were evaluated after excluding participants with unrecorded education. Tests for trend: χ^2^ = 2.76; *P* = 0.1 for vascular deaths; χ^2^ = 9.18; *P* = 0.003 for nonvascular deaths; χ^2^ = 15.09; *P* < 0.001 for all deaths.

There were 2,257 participants who died during follow-up, including 749 from a definitely vascular cause (19/1,000 ppy), 1,280 from a definitely nonvascular cause (32/1,000 ppy), and 228 (6/1,000 ppy) from an unknown cause. Across levels of education, there was a significant trend toward increased risk of vascular death (χ^2^ = 14.23; *P* < 0.001), nonvascular death (χ^2^ = 29.42; *P* < 0.001), and all-cause death (χ^2^ = 51.52; *P* < 0.001; Fig 2A) with lower educational attainment. All-cause mortality was twice as high among those with no formal education compared with tertiary-educated participants (RR, 2.05; 95% CI, 1.62-2.58), with significant increases seen for both vascular (RR, 1.84; 95% CI, 1.21-2.81) and nonvascular (RR, 2.15; 95% CI, 1.60-2.89) causes of death (Fig 2A). After adjustment for potential effect mediators, trend χ^2^ test results decreased by more than half (vascular death: χ^2^ = 2.76; *P* = 0.1; nonvascular death: χ^2^ = 9.18; *P* = 0.002; and all-cause death: χ^2^ = 15.09; *P* < 0.001, respectively; Fig 2B).

Of the 1,280 nonvascular deaths during the study, 337 (26%) were due to kidney failure; 278 (22%) to cancer; 224 (18%) to respiratory causes; and 441 (34%) to other causes. Deaths due to kidney failure, cancer, and respiratory causes were inversely associated with highest educational attainment (test for trend across education levels: χ^2^ = 10.24; *P* = 0.001; χ^2^ = 6.08; *P* = 0.01; and χ^2^ = 9.24; *P* = 0.002, respectively; Fig S2). There was no trend for the outcome of incident cancer or death from cancer across education levels (trend χ^2^ = 0.94; *P* = 0.3), but there was a trend across education levels for respiratory event or death from respiratory disease (trend χ^2^ = 30.73; *P* < 0.001; Fig S3).

### Progression of CKD

**Figure 3:**
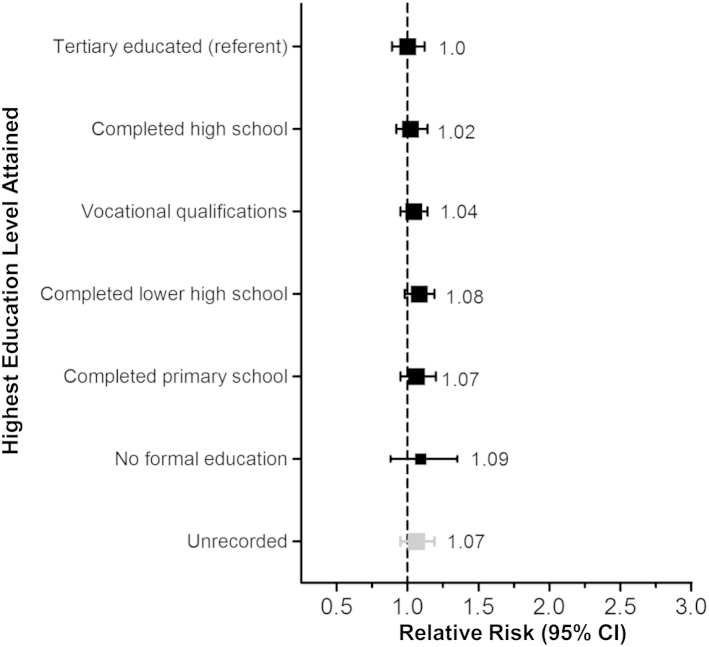
Relevance of highest education level attained among 6,245 participants not on dialysis therapy at randomization to progression to end-stage renal disease or doubling of creatinine level. Participants with end point, n = 2,446. Test for trend, Wald χ^2^ = 0.71; *P* = 0.4. Cox proportional hazards model stratified by country and adjusted for age, sex, black ethnicity, and study treatment assignment. The size of the square representing a relative risk is proportional to its inverse variance; error bars represent 95% confidence intervals (CIs). Tests for trend in all models were evaluated after excluding participants with unrecorded education.

Among 6,245 participants not receiving dialysis at baseline, 2,446 progressed to ESRD or their creatinine level doubled during follow-up (118/1,000 ppy). However, there was no significant association between highest educational attainment and this composite kidney disease outcome (trend χ^2^ = 0.71; *P* = 0.4; Fig 3). Similarly, there was no association between highest educational attainment and CKD progression at any CKD stage (Fig S4).

Among participants not receiving dialysis at baseline, the mean annual rate of decline in eGFR was 2.19 mL/min/1.73 m^2^. A strong inverse association between baseline eGFR and rate of decline was observed (ie, participants with a higher eGFR progressed more slowly; Fig S5). However, the rate of decline did not differ significantly across education levels overall or in any subgroup of CKD stage (all *P* values for trend not statistically significant).

Among the 6,245 participants not receiving dialysis at baseline, 2,994 participants progressed to ESRD or died during follow-up. There was a significant trend toward increased risk of ESRD or death across levels of educational attainment (χ^2^ = 11.27; *P* < 0.001; Fig S6A). Participants with no formal education had 32% higher risk of ESRD or death compared with tertiary-educated participants (RR, 1.32; 95% CI, 1.08-1.62). Following adjustment for potential effect mediators, the trend attenuated and was no longer statistically significant (χ^2^ = 2.59; *P* = 0.1; Fig S6B).

Associations between education level and health outcomes did not differ by randomized treatment assignment (ie, there were nonsignificant tests for interactions), except for progression to ESRD or doubling of creatinine level (*P* = 0.02).

### Health Behaviors

There was a higher likelihood of being a current smoker at baseline among people with lower educational attainment (test for trend χ^2^ = 98.09; *P* < 0.001). Furthermore, of 4,515 participants who had ever smoked, participants with higher educational attainment were more likely to have quit smoking than those with lower educational attainment (χ^2^ = 48.00; *P* < 0.001; Table S2). Lower educational attainment was associated with higher probability of obesity (ie, BMI ≥ 30 kg/m^2^ [χ^2^ = 5.13; *P* = 0.02]), but was associated with lower baseline alcohol use (χ^2^ = 29.46; *P* < 0.001). Among 8,757 participants with data for adherence to study medication at 12 months, there was no statistically significant trend across levels of educational attainment (χ^2^ = 0.90; *P* = 0.3).

## Discussion

In this study, which to our knowledge is the first large study of the relevance of educational attainment in moderate to severe CKD, we observed consistent and significant gradients in vascular risk and mortality across 6 education levels. These gradients were substantially attenuated following adjustment for available effect mediators (ie, lifestyle factors, comorbid conditions, and CKD stage). Educational level was not associated with progression of CKD in this population.

Important behavioral differences were observed across education groups. People with higher educational attainment were much less likely to be current smokers and, among those who are or have been smokers, were more likely to have quit smoking than those of lower educational attainment. Lower education level was associated with obesity, although this association was much weaker than for smoking. These associations are likely to affect both vascular and nonvascular mortality directly.^20^ However, alcohol consumption was more common among those with higher educational attainment. Although alcohol consumption might be a marker of “better health” (ie, consumption may decrease with deteriorating health),^21^ our data are limited in their ability to test this hypothesis. Contrary to other studies,^22^ we did not find a significant difference across education levels in adherence to study medication or a significant difference by educational attainment in proportions who withdrew from the study (including for reasons of nonadherence) between initial screening and final randomization (trend χ^2^ = 2.30; *P* = 0.1). In our analyses, the inclusion of all available likely effect mediators explains most of the effects of educational attainment on adverse health outcomes in CKD; however, separating the contribution of these potential mediators is not possible due to the complex relationships between them.

It was unknown a priori whether educational attainment would be associated with CKD progression. A greater rate of progression to ESRD in patients with high educational attainment has previously been reported,^23^ although the potential for selection bias in this small study that excluded patients at high risk of CKD progression was substantial. A second study of patients with CKD stages 3 to 4 reported no significant effect of educational attainment on progression to ESRD.^24^ However, the lack of an education gradient in CKD progression in our study was consistent across a number of outcomes and unlikely due to a competing mortality risk (ie, participants with lower educational attainment are more likely to die before progressing). First, there was no education gradient in progression to ESRD or doubling of creatinine level (Figs 3 and S4). Second, rates of decline in eGFR (a continuous marker of CKD progression with increased statistical power) were similar by stage of CKD, irrespective of educational attainment (Fig S5). Finally, there was also no evidence for trend in progression to ESRD or death from kidney failure specifically across education levels in a Cox proportional hazards analysis adjusting for age, sex, ethnicity, and study treatment assignment and stratified by country (χ^2^ = 1.72; *P* = 0.2). The marginally significant interaction in education gradient in CKD progression by study treatment assignment (*P* = 0.02) was in the absence of any overall effect of allocation to simvastatin plus ezetimibe on CKD progression in the study.^14^

Highest educational attainment was selected as the primary measure of socioeconomic status because it is less likely than other measures, such as income or employment, to have been influenced by disease at study entry (because it is usually completed in early life, before such disease emerges).^25^ Our analyses are therefore unlikely to be susceptible to reverse causality bias. Furthermore, highest educational attainment is considered the optimal indicator of socioeconomic status when the mechanism of effect on health outcomes is thought to be through knowledge and health behaviors and when an older population is studied.^25^ Household income data within 4 country-specific categories were collected; however, these data were not used in the main analyses because 33% of participants did not provide reliable income data at baseline, and income is likely to mediate the effects of educational attainment. However, in sensitivity analyses, the inclusion of household income in the residual effects models did not affect results for any of the outcomes. In a further sensitivity analysis, the tertiary and completed high school levels of educational attainment were combined to control for the possibility that access to tertiary education might be income dependent; trends across educational categories remained similar. *P* values for trend across these 5 education levels, after adjustment for age, sex, black ethnicity, and study treatment assignment and stratifying by country, were *P* < 0.001 (χ^2^ = 13.93) for vascular events, *P* = 0.001 (χ^2^ = 10.90) for vascular mortality, and *P* < 0.001 (χ^2^ = 26.79) for nonvascular mortality, suggesting that the gradients we report are unlikely to be unduly influenced by teriary-educated individuals.

Strengths of this study include its large size and inclusion of a wide range of patients with moderate to severe CKD and well-documented adverse health outcomes during the nearly 5-year median period of follow-up. Use of well-defined categories of educational attainment enabled assessment of a graded relationship across the 6 education levels, which was more powerful than the dichotomous exposure variables used in other studies.^23^, ^24^, ^26^ We used valid statistical methods to determine RRs of all outcomes and provided a clear rationale for adjustment of potential confounders, while avoiding overadjustment for potential effect mediators.

Limitations of the study include potential selection bias due to study inclusion criteria (ie, excluding people with prior major coronary disease, severe respiratory disease, or recent cancer may have prevented less educated and potentially sicker patients from participating). However, the likely impact of such bias would be that the effect of low educational attainment on mortality outcomes might be underestimated. In addition, an inverse association between risk factors and educational attainment may have been created through the deliberate selection of participants with CKD into SHARP that would not be present in an unselected population. This is a special case of “collider bias”^27^ that occurs when an outcome (eg, vascular death) shares common risk factors with developing CKD; this can result in the associations being biased toward the null. Such selection bias may explain at least in part the lack of association between education level and CKD progression in SHARP. While we attempted in our analyses to control for potential confounders and also to estimate associations before and after adjustment for potential effect mediators, it remains possible that biases due to residual confounding or due to unmeasured factors associated with both the effect mediators and outcome could still exist. Finally, further socioeconomic indicators such as participant income, employment level, or health insurance status, if available, may allow investigation of the extent to which these indicators modify the education-morbidity gradient. Further research into the causal mechanisms for the observed education-mortality gradient in people with moderate to severe CKD is needed.

The strong association between educational attainment and morbidity and mortality in moderate to severe CKD is consistent with findings in other chronic diseases (ie, heart failure, diabetes, and chronic lung disease).^28^, ^29^, ^30^, ^31^ We have shown that known risk factors, more prevalent in individuals with lower levels of education, are likely to mediate most of these effects. The implications of our study for clinical practice and policy are that, although risk factors for disease risks are similar irrespective of educational attainment, approaches to prevention might need to be tailored to be effective among people with lower levels of education (eg, literature to help smokers quit written at upper primary school level).^32^ Such targeted interventions also include improved communication from health professionals about chronic disease self-management and personal risk.^33^, ^34^ A direct link between low educational attainment and health literacy is known and recent research has highlighted different perceptions of personal risk among people with low compared to high health literacy.^35^

Low educational attainment is associated with increased cardiovascular risk and mortality for people with moderate to severe CKD. Modifiable lifestyle factors (eg, cigarette smoking) and prior diseases explained much of the observed association. Educational attainment was not associated with increased risk of CKD progression.These findings suggest that educational attainment should be taken into account when implementing interventions to reduce risk of adverse health outcomes in CKD.

## Figures and Tables

**Table 1:** Characteristics of the 9,270 SHARP Participants by Highest Education Level Attained at Study Entry

Characteristic at Study Entry	Tertiary Education (n = 1,062)	High School (n = 1,271)	Vocational Qualifications (n = 2,127)	Lower High School (n = 1,754)	Primary School (n = 1,378)	No Formal Education (n = 347)	Education Unrecorded (n = 1,331)	*P* Values^a^ for Heterogeneity or Trend^b^ Across Education Levels
Age, y	61 ± 12	59 ± 12	61 ± 12	60 ± 12	63 ± 12	67 ± 11	62 ± 12	<0.001
Sex								<0.001
Male	73	67	67	55	57	44	63	
Female	27	33	33	45	43	56	37	
Ethnicity								<0.001
White	75	60	88	70	54	43	83	
Black	4	8	1	2	1	3	2	
Asian	19	29	9	26	40	51	10	
Other	2	3	2	2	5	4	5	
Region								<0.001
Europe	54	42	78	56	34	42	61	
North America	16	19	5	8	8	3	9	
Australia/NZ	16	12	8	14	19	6	22	
China	9	14	7	14	13	22	<1	
Southeast Asia	5	13	2	8	26	27	8	
Smoking^c^								
Never	63	53	51	48	45	47	53	0.002^b^
Former	32	35	37	37	36	31	34	0.06^b^
Current	6	11	12	15	20	23	12	<0.001^b^
Current alcohol use^c^	36	28	29	25	18	14	27	<0.001^b^
BMI, kg/m^2,^^c^	26.5 ± 5.5	26.9 ± 5.6	27.4 ± 5.8	27.5 ± 5.5	27.2 ± 5.7	26.9 ± 5.6	26.8 ± 5.7	<0.001^b^
History of diabetes mellitus^c^	19	21	24	23	25	22	21	<0.001^b^
History of vascular disease^c^	11	14	16	16	18	17	14	0.008^b^
Albumin, g/dL^c^	4.03 ± 0.34	4.03 ± 0.42	4.01 ± 0.34	3.99 ± 0.40	3.99 ± 0.37	3.97 ± 0.42	4.01 ± 0.35	0.002^b^
Hemoglobin, g/dL^c^	12.1 ± 2.1	12.2 ± 2.1	12.1 ± 2.3	11.9 ± 2.1	11.9 ± 2.2	11.9 ± 2.0	12.1 ± 2.2	<0.001^b^
Renal diagnosis^c^								
Diabetic nephropathy	12	14	15	15	17	12	13	
Cystic kidney disease	16	12	11	10	8	10	11	
Other^d^	71	74	74	74	74	77	76	
ln(UACR), mg/g^c^	5.0 ± 1.8	5.2 ± 1.8	5.3 ± 1.9	5.3 ± 1.8	5.4 ± 1.9	5.4 ± 1.8	5.3 ± 1.8	0.001^b^
Systolic BP, mm Hg^c^	137 ± 22	138 ± 22	140 ± 23	139 ± 22	142 ± 22	141 ± 22	138 ± 23	<0.001^b^
Diastolic BP, mm Hg^c^	79 ± 13	79 ± 13	79 ± 13	79 ± 12	79 ± 13	80 ± 13	78 ± 13	0.4^b^
Total cholesterol, mmol/L^c^	4.91 ± 1.1	4.92 ± 1.2	4.89 ± 1.2	4.90 ± 1.1	4.81 ± 1.2	4.71 ± 1.2	4.90 ± 1.2	0.03^b^
LDL cholesterol, mmol/L^c^	2.79 ± 0.9	2.80 ± 0.9	2.77 ± 0.9	2.81 ± 0.9	2.73 ± 0.9	2.67 ± 0.9	2.76 ± 0.9	0.03^b^
eGFR, mL/min/1.73 m^2,^^c^^e^	26.0 ± 12.7	25.6 ± 12.8	25.0 ± 12.8	25.0 ± 12.8	24.8 ± 13.0	24.2 ± 12.8	24.7 ± 12.7	0.07^b^
CKD stage^c^								
3^f^	25	23	23	22	19	17	20	<0.001
4	32	31	32	29	27	30	28	
5, not on RRT	15	16	15	16	16	16	15	
RRT	27	30	29	33	39	38	36	

*Note:* Values for categorical variables are given as percentage; for continuous variables, as mean ± standard deviation. There were no significant differences in allocation to study medication (simvastatin plus ezetimibe vs placebo) across education levels, Pearson χ^2^ = 2.28; *P* = 0.9. The subgroups of highest educational attainment are mutually exclusive.

Abbreviations and definitions: BMI, body mass index; BP, blood pressure; CKD, chronic kidney disease stage calculated using the CKD-EPI (CKD Epidemiology Collaboration) creatinine equation^13^; eGFR, estimated glomerular filtration rate; NZ, New Zealand; RRT, renal replacement therapy; SHARP, Study of Heart and Renal Protection; UACR, urinary albumin-creatinine ratio.

a *P* values are calculated using likelihood ratio test statistics from linear regression models for continuous baseline variables and logistic regression models for categorical baseline variables, with further adjustment for age, sex, ethnicity, and country.^18^

b In tests for trend, the highest education category is coded as 1, next highest as 2, and so on; education unrecorded category is excluded. The difference in minus twice the log likelihood statistic between nested models that include and exclude this education term was used to obtain a trend *P* value.

c Adjusted for age, sex, black ethnicity, study treatment allocation, and country.

d Other renal diagnosis includes glomerulonephritis, n = 1,723; hypertensive disease, n = 1,397; pyelonephritis, n = 607; and other known and unknown disease, n = 2,823.

e For patients not on dialysis therapy.

f Predominantly CKD stage 3b patients.

## Supplementary Material

Supplementary Table S1 (PDF) Highest educational attainment as defined in countries participating in SHARP.

Supplementary Table S2 (PDF) Logistic regression results showing relevance of highest education attained to health behaviors.

Supplementary Figure S1 (PDF) Assumed relationships between highest education attained, baseline characteristics, and health outcomes.

Supplementary Figure S2 (PDF) Relevance of highest education attained to cause-specific nonvascular mortality.

Supplementary Figure S3 (PDF) Relevance of highest education attained to fatal and nonfatal nonvascular events.

Supplementary Figure S4 (PDF) Relevance of highest education attained to risk of progression to ESRD or creatinine doubling.

Supplementary Figure S5 (PDF) Relevance of highest education attained to annual rate of change in eGFR.

Supplementary Figure S6 (PDF) Relevance of highest education attained to risk of progression to ESRD or death.

Supplementary Item S1 (PDF) Definition of vascular event outcome.

Supplementary Item S2 (PDF) Definitions for model covariates.
