# Supplementary material for: Impact of Educational Attainment on Health Outcomes in Moderate to Severe CKD
Source: Am J Kidney Dis. 2016 Jan;67(1):31–9. doi: 10.1053/j.ajkd.2015.07.021 (PMC4685934; doi:10.1053/j.ajkd.2015.07.021)
Supplement: Supplementary Table S1 (PDF) — Highest educational attainment as defined in countries participating in SHARP. [file mmc1.pdf]

Table S1: Highest educational attainment as defined in countries participating in SHARP

Country	Highest education level: Tertiary		Completed high school	Vocational qualifications	Completed lower high school	Completed primary school	No formal education
**UK**	Post graduate qualification (PhD, MA)	Bachelor degree or equivalent (BA, BSc)	A level or equivalent	Vocational qualifications (GNVQ/GSVQ/NVQ/ SVQ, apprenticeship)	GCSE / O level/CSE	Did not complete secondary education	No formal education or less than one year of formal education
**Australia**	Post graduate qualification (PhD, MA)	Bachelor degree or equivalent (BA, BSc)	Higher School certificate or leaving certificate	Vocational course or apprenticeship	School certificate or Intermediate certificate	Did not complete secondary education	No formal education or less than one year of formal education
**New Zealand**	Post graduate qualification (PhD, MA)	Bachelor degree or equivalent (BA, BSc)	Higher School certificate, 6th form or University Entrance qualification	Vocational course or apprenticeship	School certificate	Did not complete secondary education	No formal education or less than one year of formal education
**Malaysia**	Post graduate qualification	Bachelor degree or equivalent	Completed secondary education	Vocational course or apprenticeship	Some but did not complete secondary education	Primary education	No formal education or less than one year of formal education
**Thailand**	Post graduate qualification	Bachelor degree or equivalent	Upper secondary education (6 years)	Vocational qualifications	Lower secondary education (3 years)	Primary education	No formal education or less than one year of formal education
**USA**	Master degree, professional degree or PhD	Bachelor degree or equivalent (BA, BS)	High school diploma/ High school equivalency diploma or GED Award	Vocational certificate or vocational degree	Secondary education (grades 7-9)	Did not complete secondary education	No formal education or less than one year of formal education
**Canada**	Post graduate qualification of professional degree (PhD, MA)	Bachelor degree or equivalent (BA, BSc)	General high school graduation diploma or equivalent	Vocational certificate or diploma	Secondary school diploma or certificate	Did not complete secondary education	No formal education or less than one year of formal education
**Germany (in German)**	Promotion	Universitätsabschluss	Abitur; allgemeine Hochschulreife; Fachabitur	Berufschulabschluss	Realschulabschluss, mittlere Reife, Hauptschulabschluss	Ohne Hauptschulabschluss	Keine Grundschule oder weniger als 1 Jahr Grundschule
**Austria (in German)**	Doktorat	Universitatdiplom	Matura, Reifeprufung	Abschluss einer technischen Schule oder Berufschule	Sekundarschule bis zur 8.Klasse (Hauptschulabschluss)	Ohne Hauptschulabschluss	Keine Grundschule oder weniger als 1 Jahr Grundschule
**Norway**	Tertiary education (2nd degree or doctorate)	Tertiary education (1st degree)	Upper secondary education (Vitnemal)	Vocational qualifications	Lower secondary (Grunnskolens Avgangsvitenal)	Did not complete secondary education	No formal education or less than one year of formal education
**Sweden**	Post graduate qualification	Bachelor degree, university diploma or equivalent	Completed upper secondary education (Slutbetyg fran gymnasieskolan)	Vocational qualifications	Compulsory basic school (slutbetyg fran grundskolan)	Did not complete secondary education	No formal education or less than one year of formal education
**Finland**	Post graduate qualification	Bachelor degree, Polytechnic degree or equivalent	Upper secondary school (Matriculation Examination)	Vocational qualifications	Lower secondary school (Leaving certificate)	Did not complete secondary education	No formal education or less than one year of formal education

**Denmark**	Tertiary education (2nd degree or doctorate)	Tertiary education (1st degree)	Upper secondary education of Gymnasium (e.g. Studentereksamen)	Primary vocational qualifications	Lower secondary education	Did not complete secondary education	No formal education or less than one year of formal education
**France**	Post graduate qualification (License, Maitrise, DESS, DEA, Doctorate)	Undergraduate Diploma (DEUG, DEUST)	Le Baccalaureat	Vocational qualifications (Educational professionelle)	Secondary school certificate (Brevet des colleges)	Did not complete secondary education (Ecole secondaire)	No formal education or less than one year of formal education
**Netherlands**	Post graduate qualification (Doctorandus, meester and doctor equivalent)	University degree, HBO or equivalent (Ingenieur, baccalaureus)	Upper secondary general program or equivalent (HAVO, VWO)	Vocational training qualifications (Beroepsnderwijs)	Junior secondary general program or equivalent (MAVO)	Did not complete secondary education (Ecole secondaire)	No formal education or less than one year of formal education
**Czech Republic**	Post graduate qualification (Magisterske or Doktorske)	Bachelor degree or university degree equivalent	Gymnasium certificate or equivalent	Vocational and general training or equivalent	Completion of basic school level or equivalent	Did not complete secondary education	No formal education or less than one year of formal education
**Poland**	Post graduate qualification (Certificate, Magister degree or Doctor equivalent)	Professional degree or university studies equivalent	Secondary general school certificate or equivalent	Basic vocational training or equivalent	Matura certificate or equivalent	Did not complete secondary education	No formal education or less than one year of formal education
**China**	Post graduate qualification (PhD, Masters)	Bachelor degree or equivalent	Technical school / vocational training or equivalent	High School	Middle School	Primary School	No formal education or less than one year of formal education
