# Supplementary material for: Impact of Educational Attainment on Health Outcomes in Moderate to Severe CKD
Source: Am J Kidney Dis. 2016 Jan;67(1):31–9. doi: 10.1053/j.ajkd.2015.07.021 (PMC4685934; doi:10.1053/j.ajkd.2015.07.021)
Supplement: Supplementary Table S2 (PDF) — Logistic regression results showing relevance of highest education attained to health behaviors. [file mmc2.pdf]

Table S2: Logistic regression results showing the relevance of highest education level attained to health behaviours

	**Lifestyle factors / health behaviours**				
**Highest education level**	**Smoker at baseline (yes/no)**	**Quit smoking (i.e. former smoker among ever smokers) at baseline (yes/no)**	**Obesity (i.e. BMI ≥30 kg/m^2) at baseline**	**Alcohol use at baseline (yes/no)**	**Adherence to study medication at 12 months**
	OR (95%CI)[a]	OR (95%CI)	OR (95%CI)	OR (95%CI)	OR (95%CI)
Tertiary	referent	referent	referent	referent	referent
Completed high school	1.93 (1.45-2.58)	0.66 (0.48-0.91)	1.35 (0.99-1.81)	0.59 (0.44-0.80)	0.56 (0.70-1.07)
Vocational qualifications	2.03 (1.54-2.66)	0.63 (0.47-0.85)	1.41 (1.01-1.94)	0.62 (0.45-0.86)	0.92 (0.75-1.12)
Completed lower high school	2.56 (1.94-3.35)	0.56 (0.42-0.76)	1.62 (1.20-2.19)	0.56 (0.42-0.77)	0.88 (0.72-1.07)
Completed primary school	3.76 (2.82-5.01)	0.37 (0.27-0.52)	1.38 (1.03-1.88)	0.36 (0.26-0.50)	0.88 (0.71-1.09)
No formal education	4.47 (3.00-6.68)	0.23 (0.15-0.38)	1.44 (0.87-2.39)	0.46 (0.23-0.92)	0.85 (0.62-1.19)
Education unrecorded	2.10 (1.58-2.80)	0.55 (0.40-0.76)	1.24 (0.91-1.69)	0.56 (0.41-0.76)	0.72 (0.58-0.89)
**Test for trend across education levels**	χ^2=98.09, p<.001	χ^2=48.00, p<.001	χ^2=5.13, p=.02	χ^2=29.46, p<.001	χ^2=0.90, p=.34

[a]All analyses adjusted for age, sex, ethnicity, country. Test for trends were evaluated in all models excluding participants with unrecorded education.
