# Supplementary material for: Impact of Educational Attainment on Health Outcomes in Moderate to Severe CKD
Source: Am J Kidney Dis. 2016 Jan;67(1):31–9. doi: 10.1053/j.ajkd.2015.07.021 (PMC4685934; doi:10.1053/j.ajkd.2015.07.021)
Supplement: Supplementary Figure S1 (PDF) — Assumed relationships between highest education attained, baseline characteristics, and health outcomes. [file mmc3.pdf]

Figure S1: Assumed relationships between highest education attained, baseline characteristics and health outcomes (simplified causal pathway diagram)

a) **Total effect**

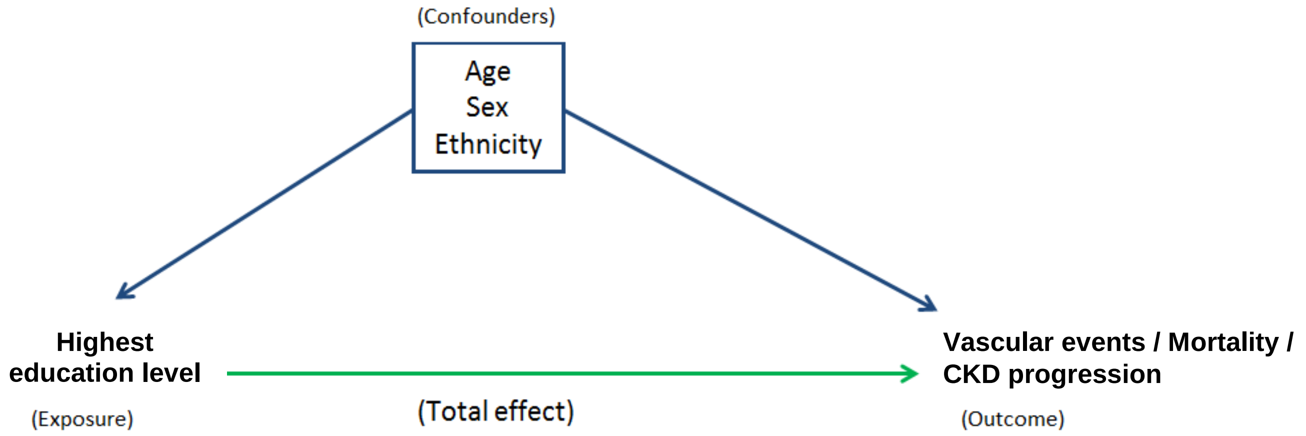

b) **Residual effect after adjustment for potential effect mediators**

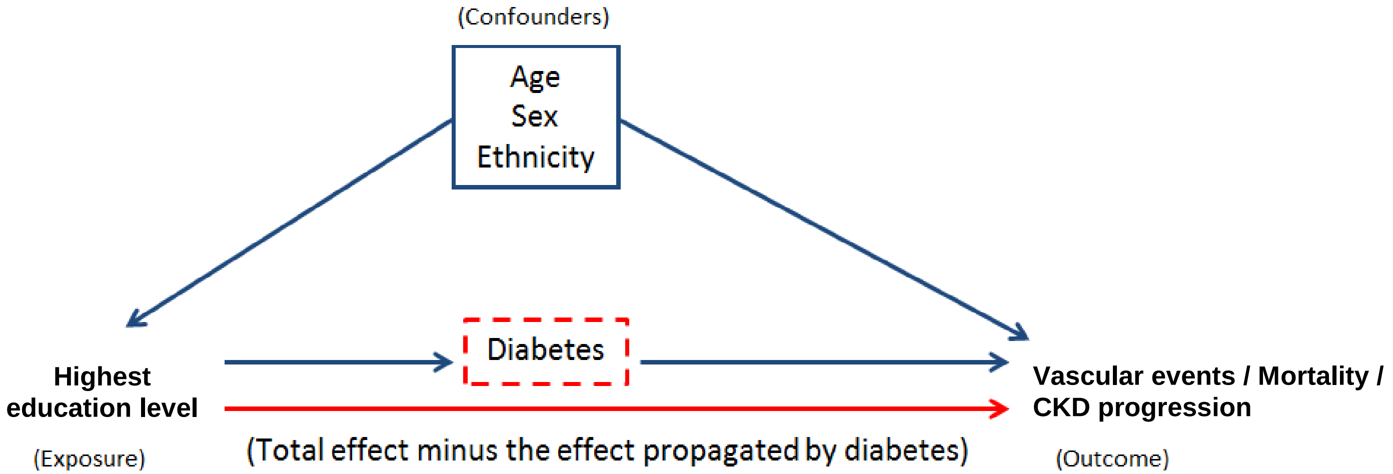

Legend: All analyses were adjusted for study treatment assignment and were stratified by country or region of participant's recruitment. Effect mediators (such as smoking, alcohol use, BMI, prior vascular disease, stage of CKD at randomisation, diabetes, cardiovascular disease, hypertension, albumin, urinary albumin: creatinine ratio, haemoglobin, cholesterol and phosphate) may lie on the causal pathway between education and vascular events, mortality or CKD progression outcomes. In order to estimate the total effect of education on outcomes these effect mediators are not adjusted for in the preferred model.
