# Supplementary material for: Impact of Educational Attainment on Health Outcomes in Moderate to Severe CKD
Source: Am J Kidney Dis. 2016 Jan;67(1):31–9. doi: 10.1053/j.ajkd.2015.07.021 (PMC4685934; doi:10.1053/j.ajkd.2015.07.021)
Supplement: Supplementary Figure S2 (PDF) — Relevance of highest education attained to cause-specific nonvascular mortality. [file mmc4.pdf]

Figure S2: Relevance of highest education attained to cause-specific nonvascular mortality

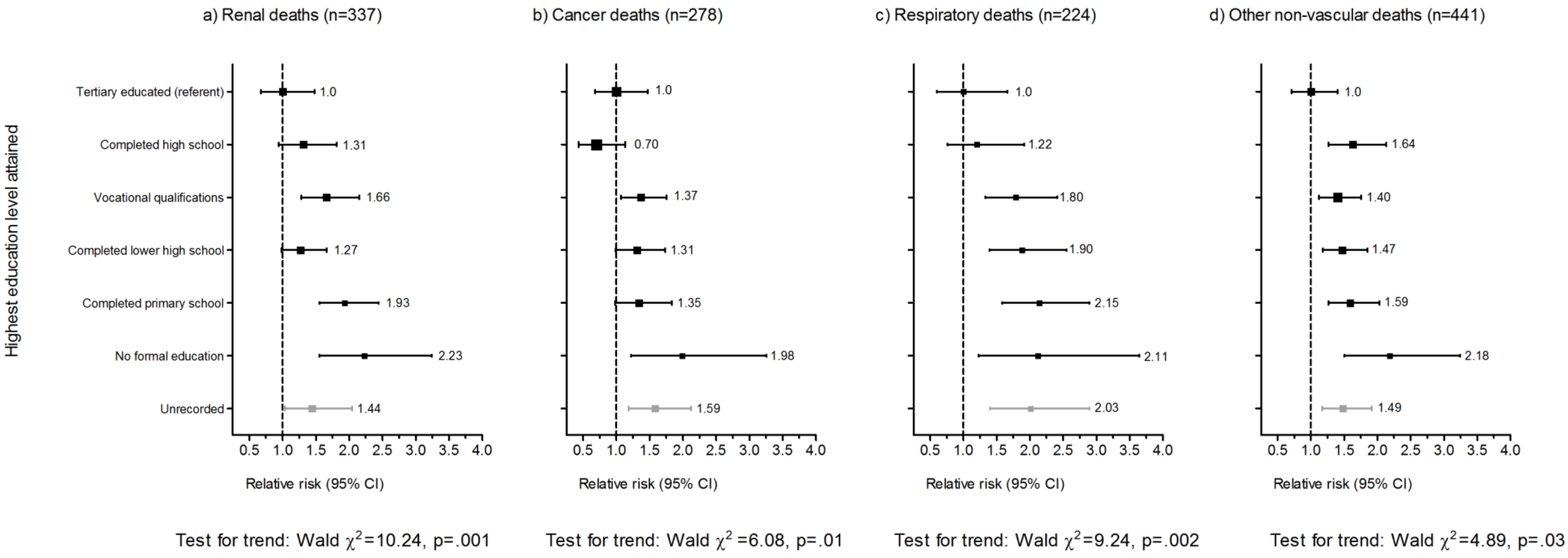

Legend: Cox proportional hazards models stratified by country and adjusted for age, sex, black ethnicity and study treatment assignment. Further adjustment of the Cox models for available likely effect mediators (smoking, alcohol use, BMI, stage of kidney disease, prior vascular disease, diabetes, renal diagnosis, systolic and diastolic blood pressure, albumin, urinary albumin:creatinine ratio; hemoglobin, phosphate, HDL cholesterol, total cholesterol) resulted in trends across education levels of χ^2=4.25, p=0.04 for Renal deaths; χ^2=1.40, p=0.24 for Cancer deaths; χ^2=2.92, p=0.09 for Respiratory deaths; and χ^2=0.89, p=.35 for Other non-vascular deaths. The size of the square representing a relative risk is proportional to its inverse variance; error bars represent 95% confidence intervals. Test for trends were evaluated in all models excluding participants with unrecorded education.
