# Supplementary material for: Impact of Educational Attainment on Health Outcomes in Moderate to Severe CKD
Source: Am J Kidney Dis. 2016 Jan;67(1):31–9. doi: 10.1053/j.ajkd.2015.07.021 (PMC4685934; doi:10.1053/j.ajkd.2015.07.021)
Supplement: Supplementary Figure S3 (PDF) — Relevance of highest education attained to fatal and nonfatal nonvascular events. [file mmc5.pdf]

Figure S3: Relevance of highest education attained to fatal and nonfatal nonvascular events events

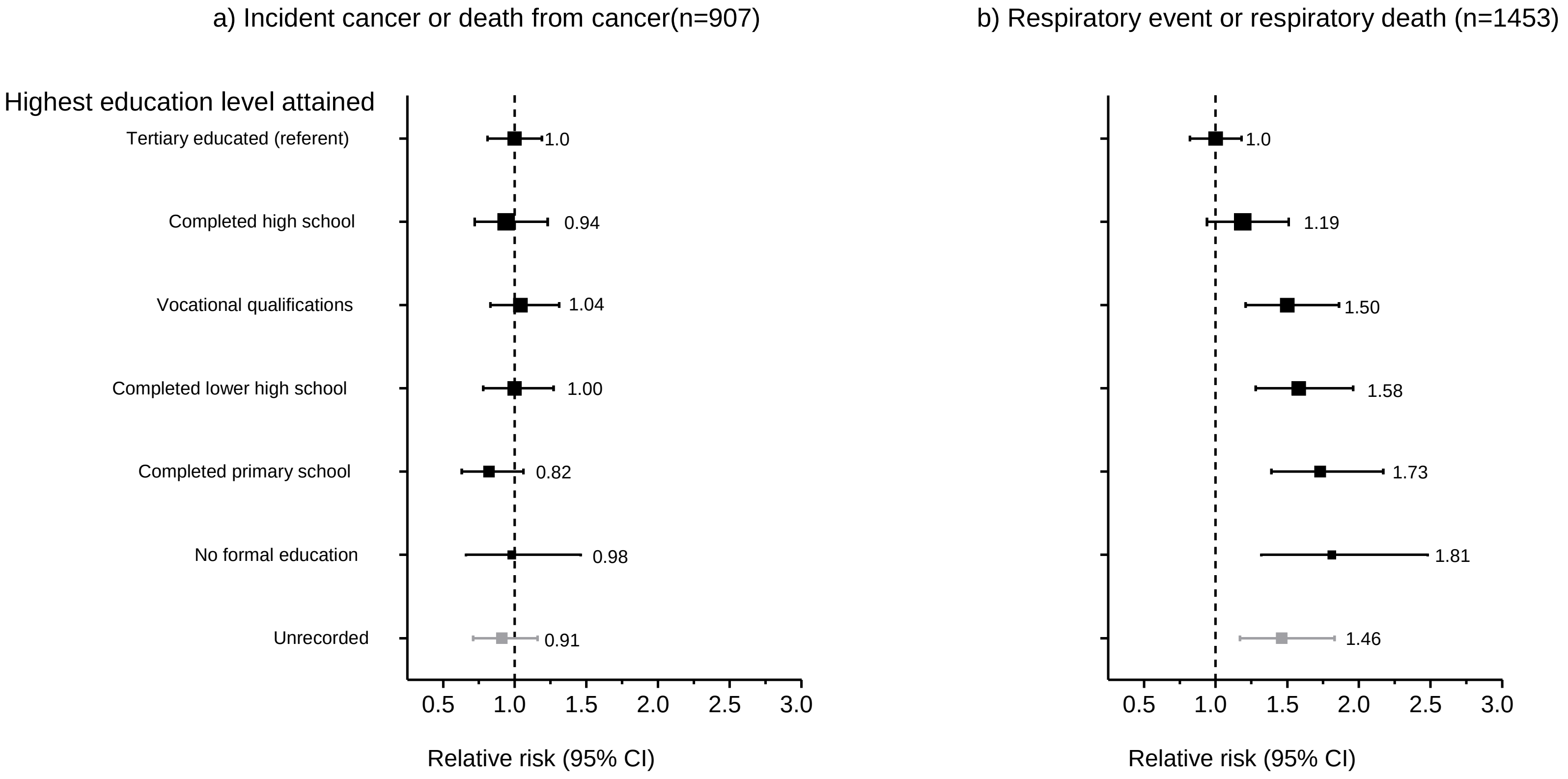

Test for trend: Wald χ^2= 0.94, p=0.3

Test for trend: Wald χ^2= 30.73, p<0.001

Legend: Model a) and b) are Cox proportional hazards models stratified by country and adjusted for age, sex, black ethnicity and study treatment assignment.. Further adjustment of the Cox models for available likely effect mediators (smoking, alcohol use, BMI, stage of kidney disease, prior vascular disease, diabetes, renal diagnosis, systolic and diastolic blood pressure, albumin, urinary albumin:creatinine ratio, hemoglobin, phosphate, HDL cholesterol, total cholesterol) resulted in trends across education levels of χ^2=1.92, p=0.17 for Cancer incidence or death; χ^2=10.68, p=0.001 for Respiratory event or death. Tests for trend were evaluated in all models excluding participants with unrecorded education.
