# Supplementary material for: Impact of Educational Attainment on Health Outcomes in Moderate to Severe CKD
Source: Am J Kidney Dis. 2016 Jan;67(1):31–9. doi: 10.1053/j.ajkd.2015.07.021 (PMC4685934; doi:10.1053/j.ajkd.2015.07.021)
Supplement: Supplementary Figure S4 (PDF) — Relevance of highest education attained to risk of progression to ESRD or creatinine doubling. [file mmc6.pdf]

Figure S4: Relevance of highest education attained to risk of progression to ESRD or doubling of creatinine level among 6,245 patients not on dialysis at randomization by baseline eGFR

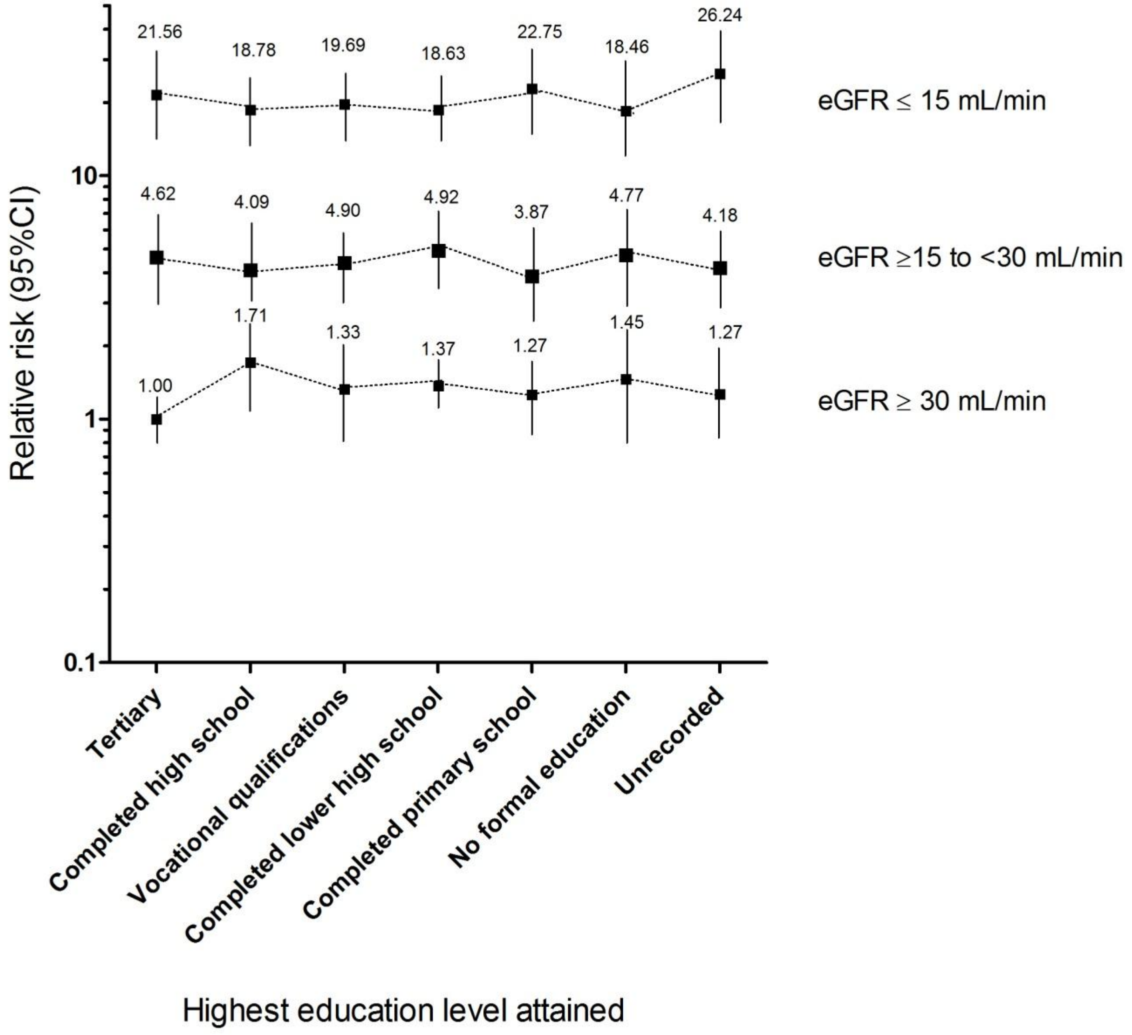

Legend: Cox proportional hazards model stratified by country and adjusted for age, sex ,black ethnicity and study treatment assignment.
The size of the square representing the relative risk is proportional to its inverse variance; error bars represent 95% confidence intervals
