# Supplementary material for: Impact of Educational Attainment on Health Outcomes in Moderate to Severe CKD
Source: Am J Kidney Dis. 2016 Jan;67(1):31–9. doi: 10.1053/j.ajkd.2015.07.021 (PMC4685934; doi:10.1053/j.ajkd.2015.07.021)
Supplement: Supplementary Figure S5 (PDF) — Relevance of highest education attained to annual rate of change in eGFR. [file mmc7.pdf]

Figure S5: Relevance of highest education attained to annual rate of change in eGFR among 6,245 patients not on dialysis at randomization, overall and by baseline eGFR

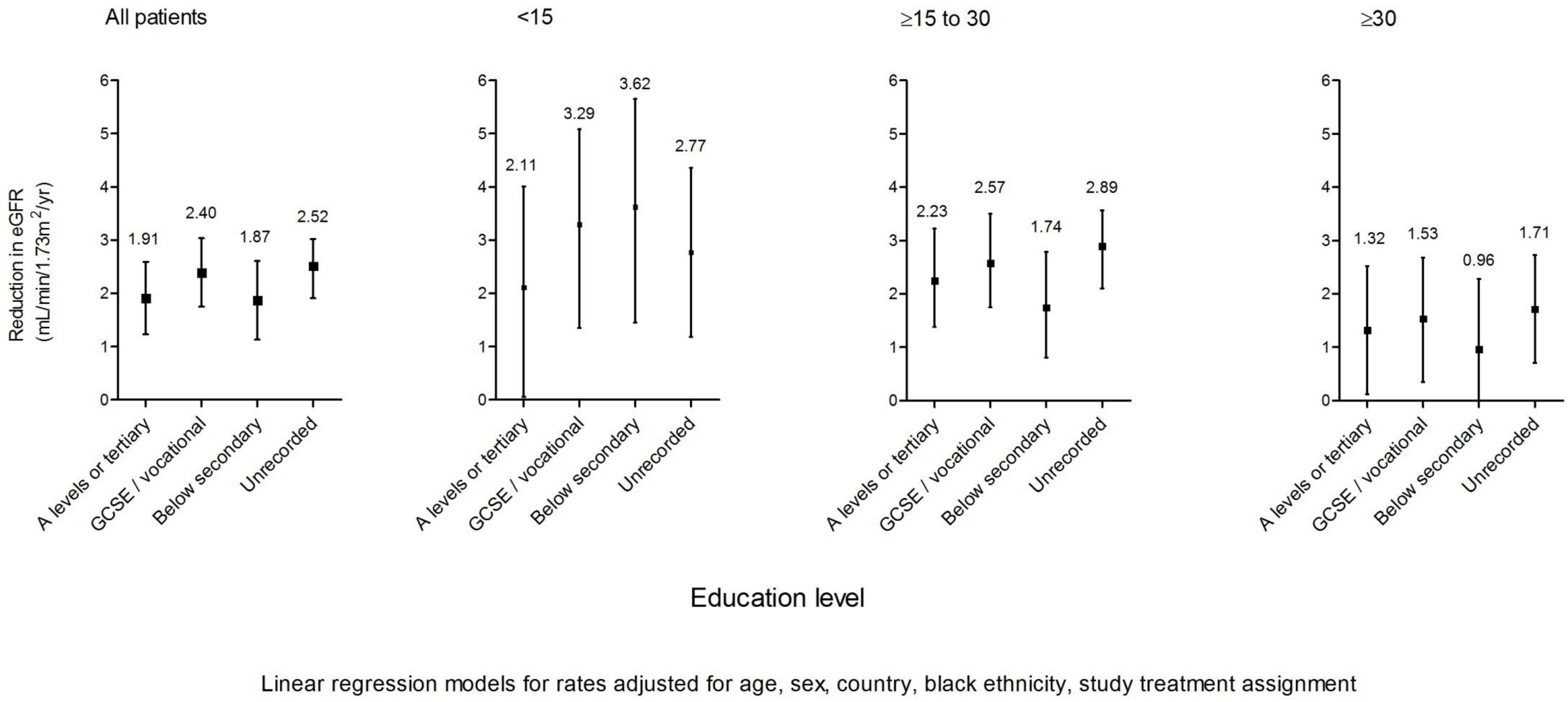

Legend: Education levels were collapsed in the following way to facilitate analysis: Tertiary + completed high school; Vocational qualifications + completed lower high school; Completed primary school + no formal education

The size of the square representing the point estimate is proportional to its inverse variance; error bars represent 95% confidence intervals
