# Supplementary material for: Impact of Educational Attainment on Health Outcomes in Moderate to Severe CKD
Source: Am J Kidney Dis. 2016 Jan;67(1):31–9. doi: 10.1053/j.ajkd.2015.07.021 (PMC4685934; doi:10.1053/j.ajkd.2015.07.021)
Supplement: Supplementary Figure S6 (PDF) — Relevance of highest education attained to risk of progression to ESRD or death. [file mmc8.pdf]

Figure S6: Relevance of highest education attained to risk of progression to ESRD or death among 6,245 participants not on dialysis at randomization

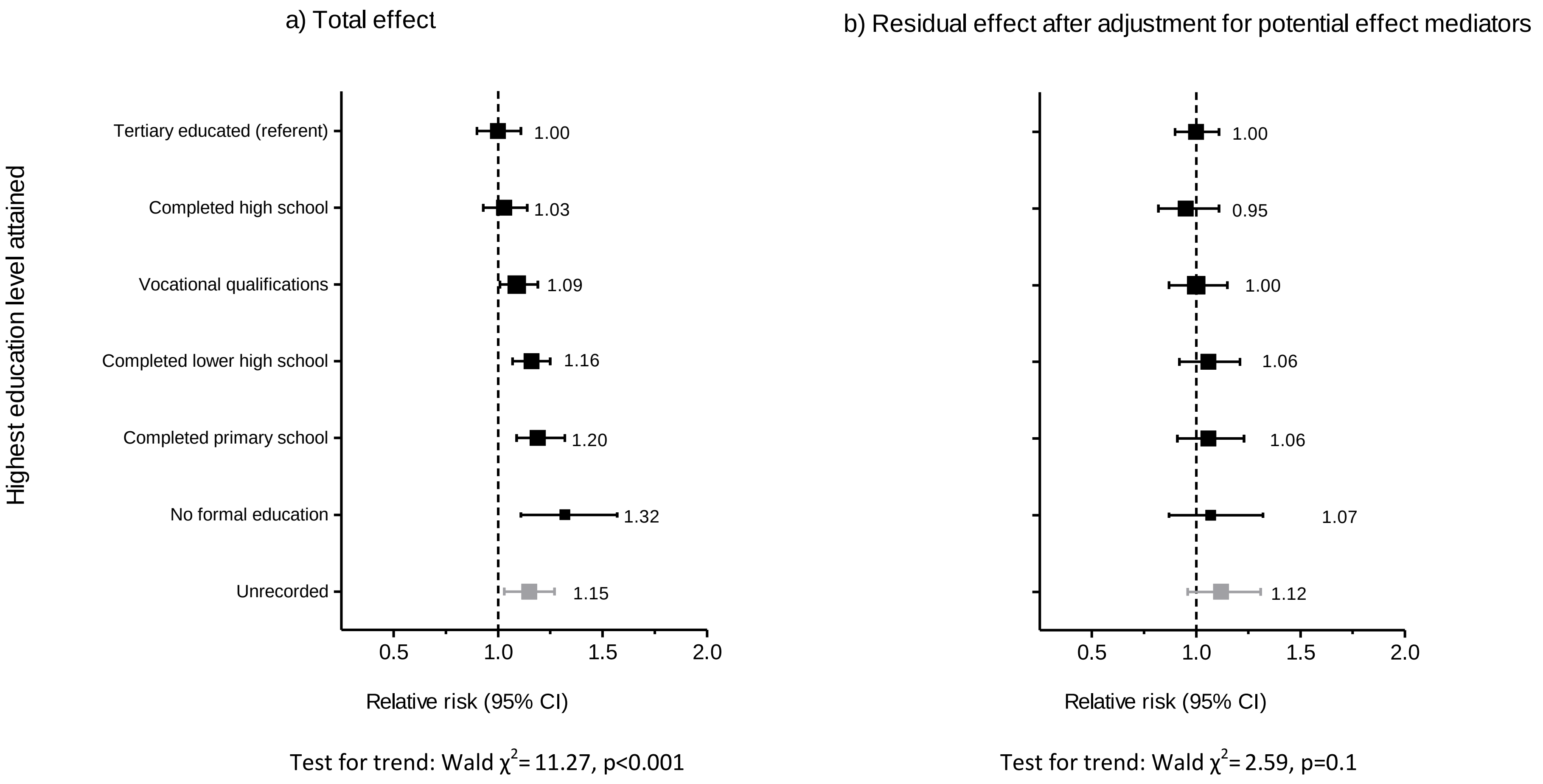

- Legend: Participants with endpoint n=2994. Cox proportional hazards model a) is stratified by country and adjusted for age, sex, black ethnicity and study treatment assignment. Model b) is stratified by country and adjusted for age, sex, black ethnicity, smoking, alcohol use, BMI, CKD stage, prior vascular disease, diabetes, renal diagnosis, systolic and diastolic blood pressure, albumin, urinary albumin:creatinine ratio, hemoglobin, phosphate, HDL cholesterol, total cholesterol. The size of the square representing a relative risk is proportional to its inverse variance; error bars represent 95% confidence intervals. Tests for trend were evaluated in all models excluding participants with unrecorded education.
