# Supplementary material for: Impact of Educational Attainment on Health Outcomes in Moderate to Severe CKD
Source: Am J Kidney Dis. 2016 Jan;67(1):31–9. doi: 10.1053/j.ajkd.2015.07.021 (PMC4685934; doi:10.1053/j.ajkd.2015.07.021)
Supplement: Supplementary Item S1 (PDF) — Definition of vascular event outcome. [file mmc9.pdf]

## Item S1: Definition of vascular event outcome

The main pre-specified vascular outcomes used in the trial included any "major atherosclerotic event" (MAE: defined as non-fatal myocardial infarction, coronary death, non-hemorrhagic stroke or arterial revascularization) and any "major vascular event" (MVE: defined as any MAE, non-coronary cardiac death or hemorrhagic stroke). To increase the number of relevant outcomes for the current epidemiologic analyses, these outcomes were expanded to include other atherosclerotic vascular events (atherosclerotic other coronary, other cerebrovascular and other peripheral arterial disease events) as well as other non-atherosclerotic vascular events not already included as a MVE (non-fatal non-ischemic heart failure, arrhythmias, valvular heart disease).

### Definitions of vascular events

Major vascular event:

- Cardiac death or non-fatal myocardial infarction
- Any stroke
- Arterial revascularization: coronary or non-coronary (excluding interventions on haemodialysis access)

Other atherosclerotic vascular event:

- Other atherosclerotic coronary events: non-fatal unstable angina, non-fatal heart failure related to ischemic heart disease
- Other atherosclerotic cerebrovascular events: transient ischaemic attack, amaurosis fugax, retinal artery occlusion
- Other atherosclerotic peripheral arterial disease:
    - aortic aneurysm (including rupture and dissection)
    - limb ischemia, limb artery embolism or thrombosis

Other non-atherosclerotic vascular event:

- Non-fatal heart failure not related to ischemic heart disease (hypertrophic cardiomyopathy, cor pulmonale)
- Non-fatal arrhythmias: atrial fibrillation, ventricular tachycardia, other tachycardia, bradycardia/heart block, other arrhythmia, cardiac arrest
- Non-fatal valvular heart disease (including valve repair or replacement)
- Non-fatal pericardial disease: pericarditis, effusion
- Subarachnoid hemorrhage
