# Supplementary material for: Impact of Educational Attainment on Health Outcomes in Moderate to Severe CKD
Source: Am J Kidney Dis. 2016 Jan;67(1):31–9. doi: 10.1053/j.ajkd.2015.07.021 (PMC4685934; doi:10.1053/j.ajkd.2015.07.021)
Supplement: Supplementary Item S2 (PDF) — Definitions for model covariates. [file mmc10.pdf]

Item S2: Definitions for model covariates

All analyses were adjusted for age, sex, ethnicity (black versus white/other), allocation to ezetimibe plus simvastatin (versus placebo) and stratified by country of recruitment. Model covariates included cigarette smoking (never, former, current), current alcohol consumption (yes/no), Body-Mass Index (BMI) (<25kg/m^2, 25-30kg/m^2, >30kg/m^2), comorbid conditions (vascular disease [yes/no], diabetes mellitus [yes/no]) and clinical characteristics (CKD stage, renal diagnosis [diabetic nephropathy, cystic kidney disease, other/unknown], systolic blood pressure [<130mmHg,130-149mmHg, ≥150mmHg], diastolic blood pressure [<75mmHg,75-84mmHg, ≥85mmHg], albumin [<39g/dL,39-40 g/dL,≥41 g/dL], urinary albumin:creatinine ratio [<30mg/g,30-299mg/g, ≥300mg/g], hemoglobin [<11.6 g/dL,11.6-12.9 g/dL,≥13 g/dL], phosphate [<1.2mg/dL,1.2-1.4 mg/dL,≥1.5 mg/dL], HDL cholesterol [<0.9 mg/dL,0.9-1.1mg/dL,≥1.2 mg/dL] and total cholesterol [<4.3mmHg,4.4-5.1mmHg,≥5.2mmHg])
